# Validation of differential DNA methylation in newborns exposed to tobacco smoke during gestation using bisulfite pyrosequencing

**DOI:** 10.17912/micropub.biology.000509

**Published:** 2022-01-07

**Authors:** Christine Crute, Yihan Liao, Esther Son, Carole Grenier, Zhiqing Huang, Cathrine Hoyo, Susan K. Murphy

**Affiliations:** 1 Integrated Toxicology and Environmental Health Program, Nicholas School of the Environment, Duke University, Durham, NC; 2 Division of Reproductive Sciences, Department of Obstetrics and Gynecology, Duke University Medical Center, Durham, NC; 3 Department of Pharmacology and Cancer Biology, Duke University Medical Center, Durham, NC; 4 University of North Carolina at Chapel Hill, Chapel Hill, NC; 5 Department of Biology, North Carolina State University, Raleigh, NC

## Abstract

Maternal exposure to tobacco smoke during pregnancy has been associated with many negative child health outcomes. Tobacco smoke exposure alters DNA methylation in the developing embryo/fetus and may be a mechanism that increases risk of later life disease. Previous studies have identified CpG sites in umbilical cord blood that are associated with *in utero* tobacco smoke exposure. We sought to validate findings for CpG sites within several of the top hit genes, *AHRR* , *CYP1A1* , and *GFI1,* using targeted quantitative bisulfite pyrosequencing. Comparing results from cord blood specimens of tobacco smoke-exposed to unexposed newborns, we confirmed significance at all previously identified CpG sites tested, including one in *AHRR* (p=0.007), three in *CYP1A1* (p<0.0001), and one in *GFI1* (p=0.008). These assays also captured novel differentially methylated CpGs located near the identified sites that were not included in the prior array-based studies (p value range, 0.02 to <0.0001). These results validate the prior findings and provide a simplified and more economical approach to analysis of CpG sites for expanded use as biomarkers of *in utero* tobacco smoke exposure.

**Figure 1:**
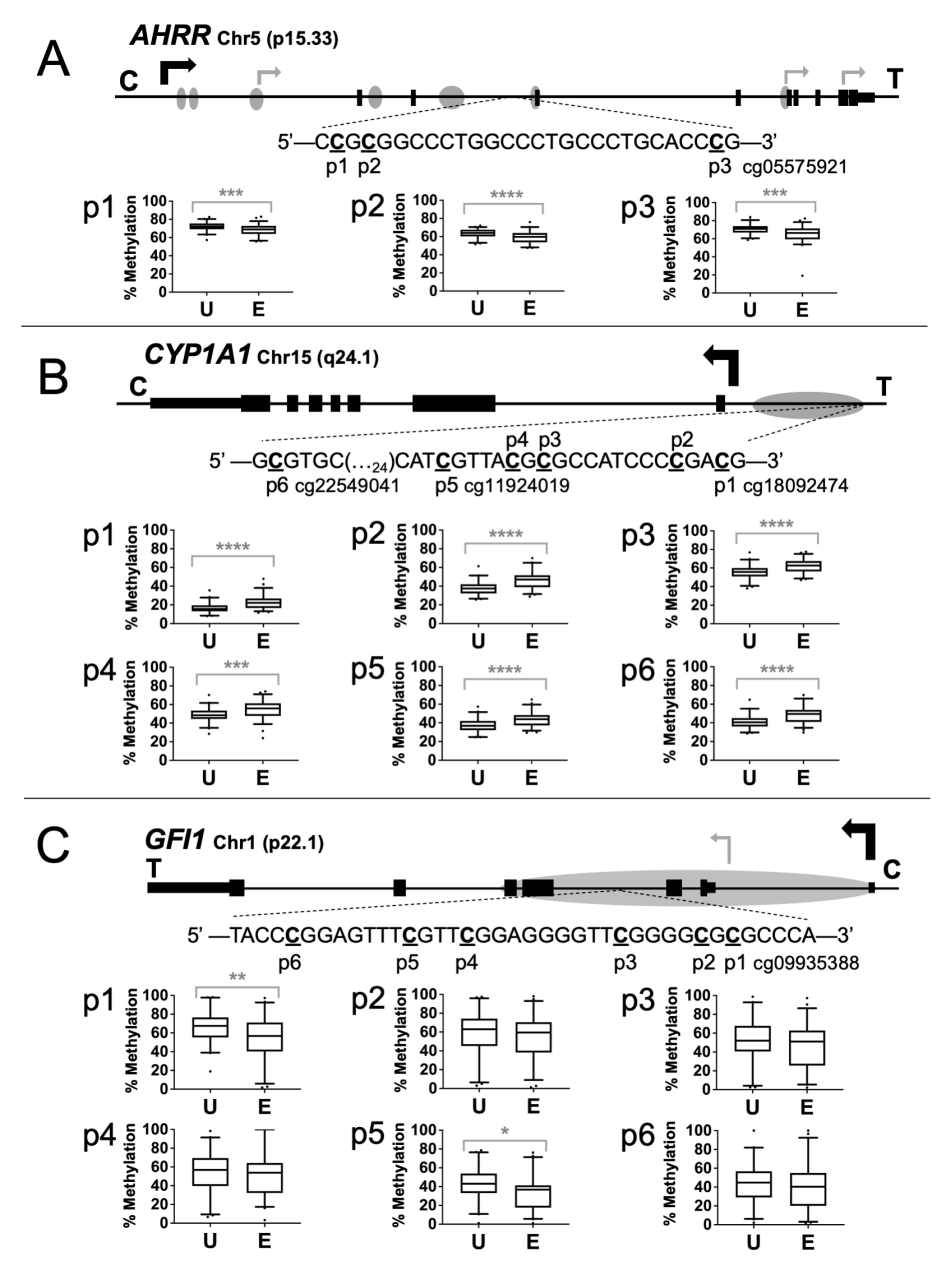
**Figure 1.** Schematics and pyrosequencing results for unexposed (U) and exposed (E) individuals for the (A) *AHRR* gene (RefSeq: NM_001242412, (B) *CYP1A1* gene (RefSeq: NM_000499) and (C) *GFI1* gene (RefSeq: NM_001127216). Illumina BeadChip probe positions are designated. Black arrows, transcription start sites; gray arrows, alternative start sites. Gray ovals, CpG islands; black rectangles, exons; shorter rectangles, UTRs. Whiskers, 5-95 percentiles. p≤0.05*, p≤0.01**,p≤0.001***, p≤0.0001****.

## Description

The Developmental Origins of Health and Disease (DOHaD) hypothesis is based on anecdotal and empirical findings that early life exposures are associated with later life susceptibility to disorders and diseases (Barker 1990). Our susceptibility to *in utero* toxicant exposures is now a major focal point in public health research. Gestational exposure to tobacco smoke is associated with low birth weight (Sbrana *et al.* 2011), airway hyperreactivity associated with asthma (Lee *et al.* 2015), and neurodevelopmental effects such as attention deficit hyperactivity disorder (Nomura *et al.* 2010; Sciberras *et al.* 2011). These outcomes have been linked to epigenetic alterations, including changes in DNA methylation (Suter *et al.* 2013; Markunas *et al.* 2014; Richmond *et al.* 2015).

A previous study utilizing the Infinium HumanMethylation450 (450k) BeadChip reported differential methylation related to *in utero* tobacco smoke exposure (Joubert *et al.* 2012) and identified 26 significant CpG probes. A number of these are in coding regions of the growth factor independent 1 transcription repressor ( *GFI1* ) and the aryl-hydrocarbon receptor repressor ( *AHRR* ) genes, in addition to a region upstream of the cytochrome P450 gene, *CYP1A1* . Further studies have validated these results using the same or similar array-based methods (Richmond *et al.* 2015; Joubert *et al.* 2016; Rotroff *et al.* 2016; Rzehak *et al.* 2016). Here we sought to validate these findings using bisulfite pyrosequencing assays to measure DNA methylation in umbilical cord blood leukocytes from infants who were unexposed or exposed to tobacco smoke during pregnancy.

The sequence and positions of the specific CpGs analyzed are presented in **Figure 1A-1C** . The 146 bp PCR amplicon for *AHRR* contains three CpGs, one of which corresponds to probe cg05575921. The 226 bp *CYP1A1* amplicon contains six CpG sites, including CpGs corresponding to probes cg22549041, cg11924019, and cg18092474. The 61 bp *GFI1* amplicon is 61 bp long and contains six CpG sites, one of which corresponds to probe cg09935388.

Pyrosequencing assays were tested in duplicate or triplicate with mixtures of Qiagen’s Epitect fully methylated and unmethylated human DNAs containing 0%, 25%, 50%, 75% or 100% methylated DNA. Measured methylation was plotted against expected methylation levels. Goodness of fit assays for each CpG showed the assays measured incremental increases in DNA methylation as the amount of input methylated DNA increased across the range of possible values ( *AHRR* : R ^2^ =0.97, p=0.0028; *CYP1A1* : R ^2^ >0.96, p=0.0029; *GFI1* : R ^2^ >0.92, p=0.003).

The assays were then used to measure methylation in human umbilical cord blood DNAs from the Newborn Epigenetics STudy (NEST) cohort. Differential methylation was substantiated for the HumanMethylation450 probe CpGs for *AHRR* cg05575921 (p=0.0007) ( **Figure 1A** ), *CYP1A1* cg11924019, cg22549041, and cg18092474 (p<0.0001) ( **Figure 1B** ), and *GFI1* cg09935388 (p=0.008) ( **Figure 1C)** *.*

An advantage of targeted analyses by pyrosequencing is the ability to assess adjacent CpG sites that may not be represented on the 450k platform. For *AHRR* , sites p1 and p2 showed an average decrease in the exposed group of 3.9% (p=0.0009) and 4.9% (p<0.0001), respectively. For *CYP1A1* , sites p2, p3, and p4 showed an average increase in the exposed newborns of 8.4% (p<0.0001), 6.7% (p<0.0001), and 6.0% (p=0.0006), respectively. The *GFI1* assay identified only one additional CpG site as significantly differentially methylated. Site p5 exhibited a 9.1% (p<0.02) decrease in the exposed group.

Using an independent quantitative method, we corroborated the findings from previously published studies of cord blood showing altered DNA methylation in association with *in utero* tobacco smoke exposure using array-based platforms (Joubert *et al.* 2012; Joehanes *et al.* 2016; Joubert *et al.* 2016; Tehranifar *et al.* 2018). We found significantly different levels of methylation between unexposed and exposed infant cord blood at identified sites for *AHRR* , *CYP1A1,* and *GFI1* . We also showed significant differences in methylation at adjacent CpG sites that were not represented on the arrays used in the prior studies. These CpGs may collectively be useful as biomarkers for *in utero* tobacco smoke exposure using a relatively cost-efficient and scalable platform.

*AHRR* and *CYP1A1* are involved with the aryl-hydrocarbon receptor (AhR) pathway, which is known to facilitate changes in gene expression after toxicant exposure. Specifically, this pathway is known to mediate toxicity of polycyclic aromatic hydrocarbons (PAHs), several of which are found in cigarette smoke (Nguyen and Bradfield 2008). Although *GFI1* has not been previously functionally associated with tobacco smoke exposure, it is involved with fundamental development processes such as hematopoiesis, inner ear and pulmonary neuroendocrine cell development, cellular proliferation, differentiation, and apoptosis, and pre-mRNA splicing control as well as immune system response (Duan *et al.* 2005; Khandanpour *et al.* 2011; Moroy and Khandanpour 2011; Joubert *et al.* 2012; Rotroff *et al.* 2016).

A limitation of the study was that we used self-reported exposure rather than cotinine levels for some cases in which these data were missing. There is a tendency for participants to underestimate exposure, which is either intentional under-reporting due to societal expectations or a lack of awareness of secondhand exposure. The overall effect of under-reporting would diminish the differences between groups, as the group classified as unexposed may contain exposed individuals.

In conclusion, we have shown concordance between the previously reported 450k BeadChip findings and those from bisulfite pyrosequencing of an independent cohort. Our results provide the foundation for implementation of these relatively low-cost assays for defined analysis of these regions in other human cohort studies seeking to define exposure effects on these vulnerable regions of the genome.

## Methods

Samples . This research was approved by the Duke Institutional Review Board (protocol Pro00043033). Cord blood specimens were selected based on self-reported smoking status during pregnancy (exposed, mother or other person living in the household smoked tobacco during her pregnancy; unexposed, mother did not smoke and was not exposed to tobacco smoke in her residence during pregnancy). These specimens were derived from the Durham, NC-based Newborn Epigenetics STudy (NEST), a mother-infant prospective cohort designed to determine the influence of early life exposures on the epigenome and health-related outcomes [see (Hoyo *et al.* 2014)].

Pregnant women were recruited from 2005 to 2011. Cord blood DNA was extracted from leukocytes separated by centrifugation from whole blood collected in vacutainer tubes containing EDTA. Samples were included in the analyses if we obtained clean PCR amplicons, the numbers of which were as follows: *AHRR* —42 unexposed, 58 exposed; *CYP1A1* —43 unexposed, 59 exposed; *GFI1* —41 unexposed, 50 exposed. Of these, maternal blood collected at enrollment for ~88% underwent measurement for cotinine, a major metabolic breakdown product of nicotine. For 12 women with missing cotinine values, self-report of tobacco exposure was used for exposure status classification. There was 87% congruence between self-reported exposure and exposure status from cotinine levels for the 820 women with plasma cotinine measurements in the parent study (Schechter *et al.* 2018).

DNA Methylation Analysis . Genomic DNA was purified using Puregene Reagents (Qiagen) and 800 ng was modified with sodium bisulfite using the Zymo EZ DNA Methylation kit (Zymo Research). Pyrosequencing assays were designed to determine methylation levels at the previously identified significant CpG sites in *AHRR* , *CYP1A1* , and *GFI1* ^14^ . All assay designs included adjacent CpGs not included on the array platform used in prior studies. Primers (Sigma) were designed using Qiagen Assay Design Software 1.0.6. *AHRR* PCR primers: F, 5’-TGG GGA TTG TTT ATT TTT GAG AG-3’; R, 5’-[biotin]AAA AAA CCC TAC CAA AAC CAC TC-3’. *CYP1A1* PCR primers: F, 5’-[biotin]ATG GGA GGT GAG GGG ATT-3’; R, 5’-CCC CAA TAC CAT TTA ACA TAA C-3’. *GFI1* PCR primers: F, 5’-AGG GAG TTA AGT GGT TAG ATA A-3’; R, 5’-[biotin] TAT ACC CAC AAC ACT CCA ATT C -3’. PCR was performed in 8-well strip tubes with total reaction volumes of 10 µL per well: 1 µL (~20 ng) BS-treated DNA and 9 µl master mix containing 5 µL PyroMark PCR buffer, 0.6 µL 25mM MgCl _2_ , 1 µL 10x Coral Load (Qiagen), 0.12 µL of both primers at 10 µM (Sigma), and 2.16 µL molecular grade water. PCR conditions were: *AHRR* , 95°C for 15 minutes; then 55 cycles of 94°C for 30s, 58°C for 30s and 72°C for 30s; final extension 72°C for 10 minutes; *CYP1A1* , 95°C for 15 minutes; then 55 cycles of 94°C for 30s, 55°C for 30s and 72°C for 30s; final extension of 72°C for 10 minutes; *GFI1* : 95°C for 15 minutes; then 5 cycles of 95°C for 30s, 66°C for 30s and 72°C for 30s; 5 cycles of 95°C for 30s, 63°C for 30s and 72°C for 30s; 5 cycles of 95°C for 30s, 60°C for 30s and 72°C for 30s; then 45 cycles for of 95°C for 30s, 57°C for 30s, and 72°C for 30s with final extension of 72°C for 10 minutes.

Sequencing primers were: *AHRR,* 5’-TTG TTT ATT TTT GAG AGG GTA-3’; *CYP1A1* , 5’- CCA AAA AAA AAA AAA TTA TAT T-3’; and *GFI1* : 5’-TTA AGT GGT TAG ATA AGG AT-3’. Validation of pyrosequencing assays was performed using EpiTect DNAs (Qiagen). Pyrosequencing was performed on a Qiagen Pyromark Q96 MD instrument and percent methylation was determined using Qiagen PyroMark CpG software 1.0.11.

Statistical analysis . All statistical analysis was conducted using GraphPad Prism version 7.03. Data from the unexposed and exposed samples were tested for normal distribution using the D’Agostino and Pearson normality test, and statistical differences between the two groups were determined using the Welch’s t-test for normally distributed data or Mann-Whitney test for data with a non-normal distribution.
